# Correlation between Epilepsy and Attention Deficit Hyperactivity Disorder: A Population-Based Cohort Study

**DOI:** 10.1371/journal.pone.0057926

**Published:** 2013-03-06

**Authors:** I-Ching Chou, Yu-Tzu Chang, Zheng-Nan Chin, Chih-Hsin Muo, Fung-Chang Sung, Huang-Tsung Kuo, Chang-Hai Tsai, Chia-Hung Kao

**Affiliations:** 1 Children’s Medical Center, China Medical University Hospital, Taichung, Taiwan; 2 Graduate Institute of Integrated Medicine, College of Chinese Medicine, China Medical University, Taichung, Taiwan; 3 Management Office for Health Data, China Medical University Hospital, Taichung, Taiwan; 4 Department of Public Health, China Medical University, Taichung, Taiwan; 5 Department of Healthcare Administration, Asia University, Taichung, Taiwan; 6 Graduate Institute of Clinical Medicine Science and School of Medicine, College of Medicine, China Medical University, Taichung, Taiwan; 7 Department of Nuclear Medicine and PET Center, China Medical University Hospital, Taichung, Taiwan; University of Utah School of Medicine, United States of America

## Abstract

**Background:**

This study presents an evaluation of the bidirectional correlation between attention deficit hyperactivity disorder (ADHD) and epilepsy using 2 cohorts from the same population database.

**Methods:**

We used data from the Taiwan National Health Insurance Research Database to establish 2 separate cohort studies with participants <19 years old. We subdivided Cohort 1 in 2 groups: (1) 2468 patients initially diagnosed with epilepsy during the period 1999–2008, and (2) 9810 randomly selected sex- and age-matched non-epileptic controls. We subdivided Cohort 2 into 2 groups: (1) 3664 patients with newly diagnosed ADHD and (2) 14 522 sex- and age-matched non-ADHD patients. We evaluated the risk of subsequent ADHD in relationship to epilepsy and vice versa in the 2 cohorts at the end of 2008.

**Results:**

The ADHD incidence in Cohort 1 was 7.76 in patients with epilepsy and 3.22 in those without epilepsy (per 1000 person-years) after a median follow-up of 7–7.5 years. The adjusted hazard ratio (HR) for ADHD was 2.54 (95% CI 2.02–3.18) in the epilepsy group compared to the non-epilepsy group. In Cohort 2, the incidence of epilepsy was 3.24 in patients with ADHD and 0.78 in those without ADHD (per 1000 person-years) after a median follow-up of 3–3.5 years and an HR of 3.94 (95% CI 2.58–6.03).

**Conclusion:**

This study shows a bidirectional association between ADHD and epilepsy in the 2 cohort studies. Causative factors may be common between these 2 disorders, leading to a cascade of transcriptional changes in the brain that alter behavior or cognition prior to seizures.

## Introduction

Epilepsy and attention deficit hyperactivity disorder (ADHD) have significant effects on the social and behavioral development of children [1]. Epilepsy is characterized by spontaneous recurrent seizures [2], but can be a pervasive condition of which seizures are one expression [3]. Similarly, ADHD has severe and persistent symptoms, such as inattention, over-activity, and impulsiveness, which initially manifest in early childhood but are associated with long-term educational and social disadvantages [4].

Previous research has indicated that ADHD is the most common disorder in preschool and school-aged children with epilepsy [5]. Behavioral difficulties in epileptic children suggest a high risk for ADHD [6], [7]. Similarly, 6.1% of ADHD children have abnormal electroencephalograph (EEG) results, compared to only 3.5% of healthy children [8]. Previous research [9], [10] on children with unprovoked seizures shows that behavioral disturbances are more common before the onset of the first seizure compared to controls. A larger study involving 148 children with first unprovoked seizures and 89 seizure-free sibling controls shows that attention problems before the first seizure, as assessed by the Child Behavior Checklist, are 2.4-fold more common in children with seizures (8.1%) than in controls (3.4%) [9].

Whether ADHD is a non-specific symptom caused by anti-epileptic drugs (AEDs), non-convulsive epileptiform discharges, or negative chronic seizure effects remains unknown. Whether ADHD is associated with epilepsy because of overlapping pathophysiologic mechanisms is also unclear. Comorbidity patterns that show shared neurobiological mechanisms involved in multiple disorders may provide useful insights.

Researchers previously conducted a two-way population-based retrospective cohort study to determine the possibility of developing schizophrenia in patients with epilepsy, and vice versa [11]. That study shows a strong bidirectional relationship between schizophrenia and epilepsy. Therefore, we hypothesize that ADHD increases the risk of subsequent epilepsy and vice versa. We conducted this study to evaluate any bidirectional correlation between ADHD and epilepsy by using 2 cohorts from the same population base.

## Materials and Methods

### Data Source

The Taiwan National Health Insurance Research Database (NHIRD), an electronic claims database of the Taiwan National Health Insurance (NHI) program, covers 99% of the 23 million population of Taiwan and contracts with more than 90% of national health care facilities [12], [13]. We analyzed an NHIRD data set of 1 million patients randomly selected from people insured in 2000. Medical claims data during the period 1996–2008 were available for all insurants. Using this data set, we obtained longitudinal person-level data on medical care usage by linking an encrypted but unique personal identification from 3 files: registry for beneficiaries, ambulatory care claims, and in-patient claims. We confirmed that all data were analyzed anonymously. This study was also approved by the Ethics Review Board at China Medical University (CMU-REC-101-012).

### Study Design and Patients

**Figure 1:**
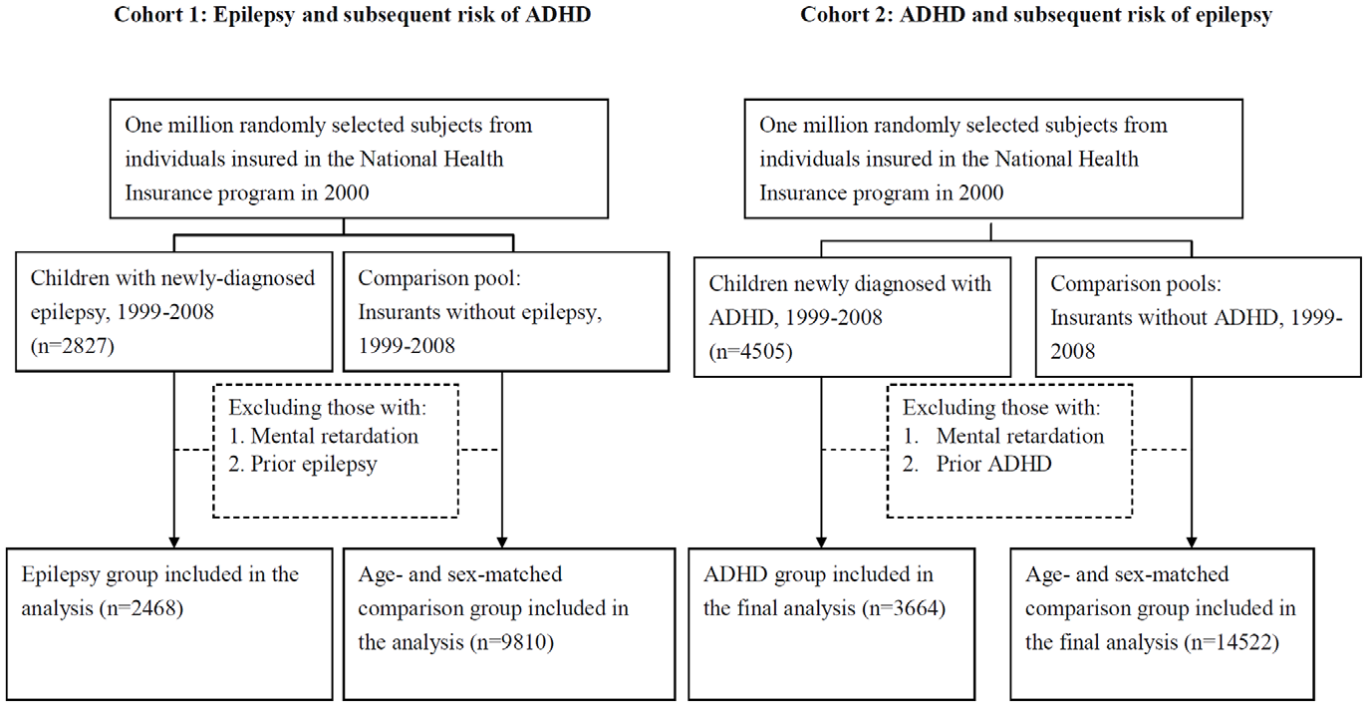
Schematic diagram of the selection of study subjects.

We conducted 2 cohort studies to assess whether epilepsy [International Classification of Disease, Ninth Revision (ICD-9), Clinical Modification, code ICD-9 345] is associated with the subsequent incidence of ADHD (ICD-9 codes 314.00, 314.01) (Cohort 1) or vice versa (Cohort 2) by using the same procedure to select the study participants (Fig. 1). The study sample consisted of children below 19 years of age without mental retardation (ICD-9 317–319). Cohort 1 included children initially diagnosed with epilepsy from 1999–2008 and a non-epileptic comparison group from the same period, excluding those with an ADHD history. Cohort 2 included children newly diagnosed with ADHD during the same period (1999–2008) and a non-ADHD comparison group, excluding those with prior epilepsy. The index date was the date of diagnosis with epilepsy (Cohort 1) or ADHD (Cohort 2). We randomly selected comparison groups in the 2 cohorts matched based on age, sex, and index year. The comparison-to-patient ratio in the group was 4.

A diagnosis of outpatient and inpatient claims revealed the onset of ADHD and epilepsy for Cohorts 1 and 2, respectively, during the follow-up period. This period started from the index date and terminated on the date of cancellation of the insurance coverage or December 31, 2008 (whichever came first).

### Statistical Analysis

We performed all statistical analyses using SAS (version 9.1 for Windows; SAS Institute Inc., Cary, NC, USA). In Cohort 1, we compared the differences in demographic characteristics, including age, sex, and urbanization level of the residential area, between patients with epilepsy and the comparison group by using chi-square and Wilcoxon two-sample tests for categorical and continuous variables, respectively. We used Cox proportional-hazards regression models to estimate hazard ratios (HRs) and their 95% confidence intervals (95% CI) for ADHD incidence in patients with epilepsy in a relationship to the comparison group. Accessibility to health care varies among geographic regions in Taiwan, requiring the adjustment of the urbanization level in the multivariate models. We repeated Cox regression analyses in subgroups stratified by age and sex. We used the same statistical analysis in Cohort 2. All significance levels were set as a two-sided *P*< .05.

## Results

### Cohort 1: Epilepsy and Subsequent Risk of ADHD

Cohort 1 included 2468 epileptic and 9810 non-epileptic children (Table 1) matched for age and sex. Among the epileptic patients, 43.8% were aged <6 years, 31.0% were 6–12 years, and all others were >12 years. The mean age (±SD) was 8.0 ± 5.3 years. Approximately 44% of the cohort comprised girls. The 2 groups showed no significant difference in the level of urbanization.

**Table 1:** Demographic characteristics of patients with and without epilepsy.

Variables	Epilepsy				*p* value*
	Without (n = 9810)		With (n = 2468)		
	n	%	n	%	
Age, years					
Mean ± SD	8±5.35		8±5.34		0.86
0–6	4274	43.6	1082	43.8	0.98
6–12	3056	31.2	766	31.0	
12–18	2480	25.3	620	25.1	
Sex					
Female	4306	43.9	1083	43.9	0.98
Male	5504	56.1	1385	56.1	
Urbanization					
1 (highest)	2653	27.0	659	26.7	0.60
2	2968	30.3	750	30.4	
3	1894	19.3	482	19.5	
4 (lowest)	2252	23.0	569	23.1	

* Chi-square test except for the *P* value of age, according to a Wilcoxon two-sample test.

During the median follow-up of 7.0 years for the epilepsy group and 7.5 years for the comparison group, the incidence of ADHD (per 1000 person-years) was 7.76 and 3.22, respectively (Table 2). Epilepsy is associated with an increased risk of subsequent ADHD (adjusted HR 2.54, 95% CI: 2.02–3.18). The age-specific HRs increased with age, at 2.26 (95% CI: 1.74–2.94), 3.53 (95% CI: 2.15–5.80), and 5.30 (95% CI: 1.42–19.78) for patients aged 0–6 years, 6–12 years, and 12–18 years, respectively. Both sexes showed an association between epilepsy and subsequent risk of ADHD.

**Table 2:** Hazard ratios for ADHD incidence with epilepsy.

	Non-epilepsy comparisongroup			Patients withepilepsy			Hazard ratio and 95% CI			
							(Patients with epilepsy vs. comparison group)			
	Cases	PY	Incidence†	Cases	PY	Incidence†	Unadjusted		Adjusted#	
All	205	63695	3.22	121	15585	7.76	2.52	(2.01–3.17)***	2.54	(2.02–3.18)***
Age, years										
0–6	159	32437	4.90	87	7944	10.95	2.26	(1.74–2.93)***	2.26	(1.74–2.94)***
6–12	34	17328	1.96	29	4236	6.85	3.50	(2.13–5.74)***	3.53	(2.15–5.80)***
12–18	4	13822	0.29	5	3405	1.47	5.13	(1.38–19.09)*	5.30	(1.42–19.78)*
Sex										
Female	34	28091	1.21	30	6949	4.32	3.59	(2.19–5.86)***	3.59	(2.20–5.86)***
Male	171	35604	4.80	91	8636	10.54	2.31	(1.79–2.99)***	2.31	(1.78–2.98)***

Note: PY, person-years at risk.

# Adjusted for age, sex and urbanization level.

† per1,000 person-years.

* *P*< .05;

** *P*< .01;

*** *P*< .0001.

### Cohort 2: ADHD and Subsequent Risk of Epilepsy

Cohort 2 included 3664 ADHD and 14 522 non-ADHD children (Table 3). More than half (64.3%) of the ADHD group was aged 6–12 years, and 22.4% were aged 0–6 years. The mean age (±SD) was 8.7 ± 3.0 years, with male predominance in the ADHD group (80.2%). The ADHD and comparison groups were matched for age and sex. Children with ADHD were more likely to reside in urbanized areas than those in the comparison group (*P* < .0001).

**Table 3:** Demographic characteristics of ADHD and non-ADHD patients.

Variables	ADHD				*P* value*
	Without (n = 14522)		With (n = 3664)		
	N	%	n	%	
Age, years					
Mean ± SD	8.7±3.0		8.7±3.0		0.66
0–6	3230	22.2	821	22.4	0.99
6–12	9348	64.4	2357	64.3	
12–18	1944	13.4	486	13.3	
Sex					
Female	2908	20.0	727	19.8	0.80
Male	11614	80.0	2937	80.2	
Urbanization					
1 (highest)	3870	26.6	1370	37.4	<0.0001
2	4311	29.7	1155	31.5	
3	2925	20.1	640	17.5	
4 (lowest)	3353	23.1	484	13.2	

* Chi-square test except for the *P* value of age, according to a Wilcoxon two-sample test.

During the median follow-up period of 3.3 years for the ADHD group and 3.5 years for the comparison group, the incidence of epilepsy (per 1000 person-years) was 3.24 and 0.78, respectively (Table 4). Children with ADHD had a 3.94-fold risk of developing epilepsy (95% CI: 2.58–6.03) after controlling for age, sex, and level of urbanization relative to the comparison group. There was an association among patient subgroups stratified by age and sex, but the HR was not significantly different among age groups and between male and female patients.

**Table 4:** Hazard ratios for the incidence of epilepsy with ADHD.

	Non-ADHD comparison group			Patients with ADHD			Hazard ratio and 95% CI			
							(Patients with schizophrenia vs. comparison group)			
	Cases	PY	Incidence†	Cases	PY	Incidence†	Unadjusted		adjusted#	
All	43	55125	0.78	44	13600	3.24	4.14	(2.72–6.31)***	3.94	(2.58–6.03)***
Age, years										
0–6	16	17395	0.92	16	4294	3.73	4.09	(2.05–8.19)***	3.79	(1.88–7.62)**
6–12	20	32190	0.62	21	7942	2.64	4.20	(2.28–7.76)***	4.16	(2.24–7.74)***
12–18	7	5541	1.26	7	1364	5.13	4.07	(1.43–11.62)**	3.84	(1.32–11.14)*
Sex										
Female	10	10544	0.95	11	2581	4.26	4.47	(1.90–10.54)**	4.44	(1.86–10.61)**
Male	33	44581	0.74	33	11018	2.99	4.04	(2.49–6.54)***	3.81	(2.34–6.21)***

Note: PY, person-years at risk.

# Adjusted for age, sex and urbanization level.

† per1,000 person-years.

* *P*< .05;

** *P*< .01;

*** *P*< .0001.

## Discussion

The prevalence rates of ADHD and epilepsy in the general pediatric population are 3%–7% and 0.05%–1%, respectively [14]–[17]. The rate of the 2 occurring together is greater than that expected by chance because ADHD and seizures may be comorbid conditions. Part of this increase is a bidirectional relationship between ADHD and epilepsy. In this study, the possibility of developing ADHD among epileptic patients is higher (adjusted HR 2.54, 95% CI: 2.02–3.18). Conversely, ADHD increases the risk of subsequent epilepsy (adjusted HR 3.94, 95% CI: 2.58–6.03), and vice versa.

The complex relationship between epilepsy and ADHD remains unclear. Researchers have proposed several hypotheses on the possible pathophysiology of their comorbidity in the context of brain development, including the effects of chronic seizures, EEG epileptiform discharges, and AEDs [18], [19]. Neurodevelopmental conditions may increase the vulnerability of children to epilepsy and ADHD. In a retrospective study, Austin et al showed that attention and behavioral problems are higher in children with epilepsy compared to their siblings 6 months prior to the first diagnosed seizure. This finding is consistent with the hypothesis that the possibility of acquiring ADHD increases in epileptic children and is independent of the effects of seizures or their treatment [20]. Hesdorffer et al evaluated ADHD symptoms in children prior to the onset of seizures, showing that ADHD is significantly more common in patients with new-onset epilepsy (31%) than in healthy controls (6%). For example, ADHD antedated the diagnosis of epilepsy in 82% of cases [21].

The pathogenesis of the relationship between epilepsy and ADHD remains unknown. A frontostriatal network dysfunction is related to ADHD without epilepsy [22]–[25], whereas evidence of frontal lobe dysfunction appears in both focal-onset and the generalized-onset types of epilepsy. Thus, the frontal lobes may correlate between epilepsy and ADHD. Their comorbidity is also supported by animal models, which may shed light on the common genetic defects underlying these disorders. In one study, rats were selectively bred to test amygdala kindling (a model for temporal lobe epilepsy) speed. The same rats were assessed for ADHD-like symptoms in subsequent generations. The fast kindling rats (genetically seizure prone) were similar to humans with ADHD compared to the slow kindling rats [26]. Another study shows that seizure-induced rats simultaneously developed behavioral and physical characteristics similar to ADHD symptoms [27].

The results of this study show that common neurobiological mechanisms may be present in epilepsy and ADHD. Identifying these mechanisms is an important consideration in the clinical management of epilepsy. Further studies should investigate genetic abnormalities that, through variable penetrance, can lead to epilepsy alone, epilepsy with ADHD, or ADHD alone. The effects of epileptiform activity in different neural network functions also require better understanding.
